# A simple technique for the fabrication of P(St-BA-AA) colloidal crystal microdots on ink-jet paper

**DOI:** 10.1016/j.heliyon.2020.e04196

**Published:** 2020-06-11

**Authors:** Ikhazuagbe Hilary Ifijen, Esther Uwidia Ikhuoria

**Affiliations:** a Product Development Laboratory, Rubber Research Institute of Nigeria, P. M. B. 1049, Benin City, Nigeria; b Department of Chemistry, University of Benin, Benin City, Nigeria

**Keywords:** Materials science, Nanotechnology, Poly(styrene-butyalcrylate-acrylic acid), Photonic crystals, Monodisperse core/shell, Terpolymer, Colloidal ink, Colloidal crystal microdots

## Abstract

The generation of three-dimensional superb packages of compact hexagonal periodic assembly with multifaceted layers was demonstrated using Poly (styrene-butylcrylate-acrylic acid) on glass substrates. The synthesized P(St-BA-AA) microspheres were used to formulate a fast drying UV curable terpolymer microspheres printing ink for the generation of colloidal crystal microdots on ink-jet paper with remarkable colours. The terpolymer microspheres and their formulated terpolymer ink undergo self-assembly to form blue monochromatic and viewing angle dependent tunable colours, thus affirming the photonic nature of the generated colloidal crystal films. Unlike other printing techniques which usually make use of specialized tools, this study generated a well ordered coloured tunable assembly of spherical shaped core-shell colloidal crystal microdots on the surface of an inkjet-paper by manually writing the as-synthesized P(St-BA-AA) UV curable printing ink microdots on it. The TEM analysis showed core and shell sizes of 198/50nm and 176/30nm for P(St-BA-AA)_1_ and P(St- BA-AA)_2_ respectively. Whenever the prepared terpolymer microspheres are used within the heating and transition temperatures of 383 °C and 110 °C, their thermal stability is retained. This simple technique of generating crystals microdots on inkjet paper may find use in optical devices, security applications and other colour coating applications.

## 1 Introduction

Materials that can regulate the flow of light due to their periodic dielectric structural nature are known as Photonic crystals [1, 2]. Iridescent colours of structural origin that is based on the Bragg diffraction of light can be generated from the complete manipulation of light on photonic crystals [1]. Several types of insects such as beetles and butterflies has used a similar mechanism to beautify their skins with shiny colors without involving the use of any conventional pigments [3] and as such, considerable attention has been attracted by the iridescent colour produced from these periodic structural materials because of the bright nature of their colours, durability and environmental friendliness [4].

Photonic crystals have shown encouraging utilization in diverse areas such as biological and chemical sensors, full-colour displays, responsive optical devices, photonic papers, beautifying and coating elements, catalytic purposes and UV shield [5, 6, 7, 8, 9, 10, 11]. Low cost production of two and three-dimensional photonic crystals with wide areas and distinct shapes can be generated from colloidal self-assembly technique [12, 13, 14]. This approach habitually results in materials with ordered particles and captivating properties. One of the usual simple ways of producing photonic crystals is based on the use of polymeric colloids with self-assembly characteristics [9, 14, 15, 16, 17, 18].

Previous studies have used different techniques to assemble polymer colloidal particles into close-packed arrays. The most well-known techniques are self-assembly under the control of an outer gravitational fields, evaporation assisted self assembly (vertical deposition) and the micro-fluidic cell method [19] with vertical deposition being the most extensively utilized because it is considered as a cheaper alternative to other methods. For example, the assembly of monodispersed polymeric material such as polystyrene (PS) and poly (styrene-methylmethacrylate-acrylic acid) (P(St-MMA-AA) into two (2-D) and three-dimensional (3-D) regular periodic structures on silicon and glass substrates have been established [20, 21]. Ink-jet printing has also emerged, as an attractive technique for fabricating coloured photonic crystals on substrates especially on polythene and paper surfaces [22, 23]. This technique depends on the careful deposition of colloidal based ink on a temporary surface in the form of droplets.

The assembly of several types of polymeric colloidal printing ink on different substrates using special analytical equipments in order to obtained structural colours has been reported [24, 25], but the fabrication of a well ordered coloured tunable colloidal crystals using a UV curable P(St-BA-AA) colloidal ink on ink-jet paper substrate through a very direct and simple method has never been attempted.

This study reports on the fabrication of a fast drying P(St-BA-AA) UV curable colloidal printing ink microdots with colour tuning property on inkjet-paper substrate using a very simple and precise technique.

## 2 Experimental section

### 2.1 Materials

Styrene, butyl acrylate (BA), acrylamide (AAm), acrylic acid (AA), ammonium per-sulphate (APS), sodium dodecyl benzenesulfonate (SDBS), water-retaining agent ethylene glycol, ammonium bicarbonate, (EG), the cross linking agent N, N-methylenebisacrylamide (99%) (BIS) and initiator dimethoxy-acetophenone (DMAP). All reagents were of analytical grade and are obtained from sigma Aldrich Inc. (USA).

### 2.2 Preparation of monodispersed P(St-BA-AA) microspheres

**Figure 1:**
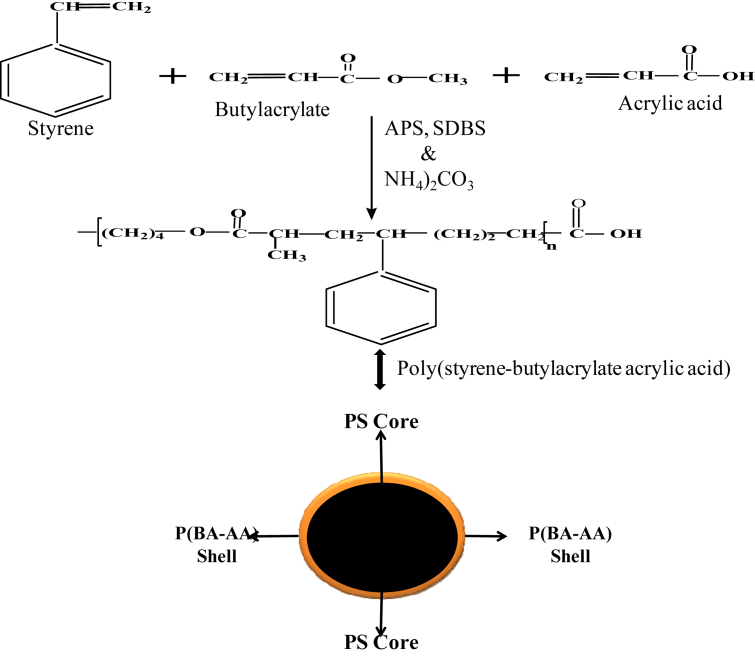
Reaction steps pointing to the development of core shell P(St-BA-AA) microspheres.

Core-shell monodispersed poly(styrene-butylacrylate-acrylic acid) (P(St-MMA-AA)) microspheres was synthesized via a batch emulsion polymerization method as described by Minghui et al. [24] with a slight alteration. In a typical experiment, 3.190g of styrene (St), 0.720g of acrylic acid (AA), 0.005g of sodium dodecylbenzene sulphonate (SDBS), and 0.085g of ammonium bicarbonate were dispersed in a 50ml two neck flask comprising 16.5g of distilled water and then stirred with a magnetic stirrer (410rpm) in the presence of nitrogen for 30minutes at a temperature of 90 °C. This was accompanied by dispersing a mix of 0.735g of butyl-acrylate (BA) monomer and 0.091g of APS initiator drop-wise inside the reaction beaker to start the polymerization process. The reaction was kept for 13 hrs at the same stirring speed and temperature. The suggested/potential reaction scheme for the synthesis of core shell P(St-BA-AA) microspheres is described in Figure 1.

### 2.3 Fabrication of photonic crystals

Photonic crystals were formed through a vertical deposition method on a glass substrate. A piece of glass slide was vertically placed in a glass vial holding the prepared colloidal P(St-BA-AA) microspheres and then diluted to 1wt % de-ionized water. This was kept in a water bath at a temperature of 55 °C. Finally, the glass substrates with the colloidal particles were transferred into a vacuum drying oven at a fixed temperature of 60 °C overnight.

### 2.4 Preparation of terpolymer colloidal ink for printing

Printing terpolymer colloidal ink was made as described by Wang et al. [26]. In a typical experiment, 2.405g of AAm, 0.0481g of BIS, 0.012g of DMAP and 4.27ml of EG was added to P(St-BA-AA) microspheres (11.1g) containing 10.91 wt% P(St-BA-AA) terpolymer microspheres and thereafter, blending in an ultrasonic bath was carried out for 15min. The formed ink was manually printed on inkjet-paper whose surface is really smooth using a pipette and then transferred to a UV chamber for 3minutes to initiate photo-polymerization. The surface of the inkjet-paper surface is very smooth.

### 2.5 Characterization techniques

Microscopic methods were adopted in determining the dimensions and morphologies of the assembled P(St-BA-AA) colloidal crystals. Scanning electron microscope (JEOL-JSM 5600 LV) was employed in carrying out the SEM imaging analysis. Multi-Mode 8 AFM equipped with NanoScope V controller (Bruker, Santa Barbara, CA, USA) was used to examine the Atomic force microscopy (AFM) imaging under anhydrous conditions at room temperature (22 ± 2 °C). FEI Tecnai 30 G2S-TWIN-TEM performed at accelerating energy of 300 kV was employed to capture the images of Transmission electron microscopic (TEM). The prepared terpolymers functional groups were ascertained using Perkin-Elmer Series Spectrum Two FT-IR spectrometer covering the wavenumber spectrum 4000-500 cm^−1^. PANalytical EMPYREAN instrument equipped with reference radiation of Cu Kα (λ = 1.54 Å) at a working voltage of 45 kV was used to determine the powder X-ray diffraction model of the synthesized P(St-BA-AA) microspheres. Thermo-gravimetric analyzer TA Q50 subjected to a nitrogen gas environment at a heat rate of 10 °C/min was used to evaluate the thermal durability of the terpolymer microspheres. Under the scattering angle of 173^o^ at 6333 nm wavelength, the average particle diameter and the distribution of sizes (polydispersity index (PDI) were estimated using Dynamic Light Scattering (DLS) (Nano-Zetasizer, Malvern Instruments) at 25 °C. The differential scanning calorimetry (DSC) measurement was measured using a DSC 2920 module in connection with the TA Instruments 5100 system at a scan rate of 10 C/min under a nitrogen atmosphere. Images of the fabricated photonic crystals were taken using Micromax 12 megapixel camera phone.

## 3 Results and discussion

### 3.1 Core-shell P(St-BA-AA) microspheres

Two pot emulsion polymerization procedure was utilized in the synthesis of monodisperse P(St-BA-AA) microspheres. A two-pot synthesis was preferred over the one-pot process because polymerization of butyl acrylate (BA) can also be initiated by the presence of heat. Due to the low emulsifier concentration (0.05g) added to the system during the synthesis, the emulsifier concentration (Ec) was therefore expected to be lower than the critical micelle concentration (CMC) [26]. This is evidence that P(St-BA-AA) colloidal particle growth took place inside the partially dissolved aqueous phase monomer rather than the micelle phase. As a result, the nucleation of the terpolymer colloidal particles was predicted to occur via homogeneous nucleation mechanism [27, 28]. The improvement made to polystyrene (PS) by the complete infiltration of butyl-acrylate/acrylic acid into the interstice of its structure introduced some level of polarity between the non-polar PS interior and the polar PBA/PAA exterior [29]. As such, the ultimate morphology of the terpolymer microspheres was accordingly anticipated to be a core-shell structure [15] (Figure 1). The P(St-BA-AA) microspheres are composed of hard PS core (internal structure) covered with thin shell rich in PAA and PBA (exterior) (Figure 1) [30].

### 3.2 Functional groups and thermal analysis of P(St-BA-AA) microspheres

**Figure 2:**
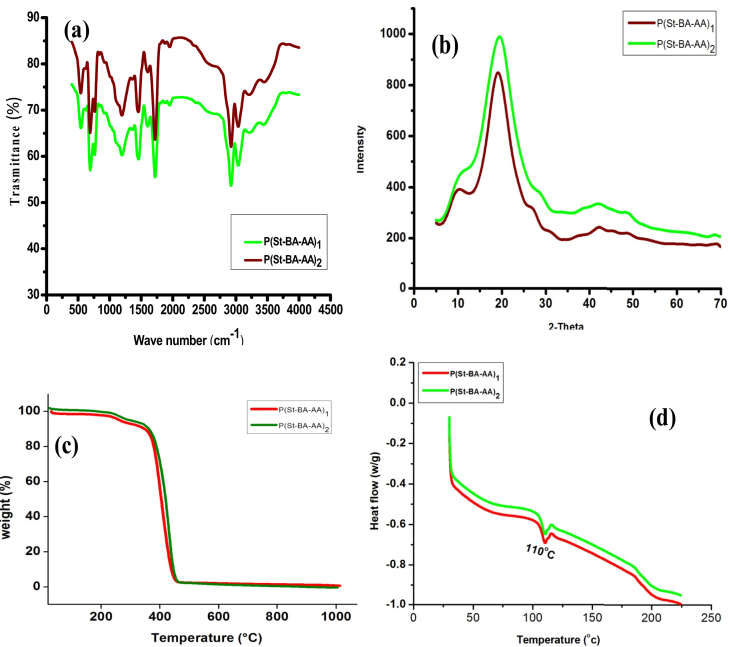
(a) FTIR spectra (b) XRD patterns (c) TGA curve (d) DSC results of P(St-BA-AA) microspheres.

Figure 2a shows the functional groups present in the as-synthesize P(St-BA-AA) compounds with different intensities. The characteristic absorbance band of the C=O group stretching vibration in carboxylic acid emerged at 1736 cm^−1^ [30]. The C–H in-plane bending vibration and C–H stretching vibration developed at 1449 cm^−1^ and 1380 cm^−1^ sequentially. The absorbance peak at 1600 cm^−1^ is due to aromatic C=C–C stretching vibration. The absorbance peaks at 762cm^−1^ and 704cm^−1^ are due to the presence of aromatic -C-H out-of-plane bend [12]. The absorbance bands at 3024 cm-^1^ and 2924 cm-^1^ are allotted to aromatic C–H and methylene groups apiece [31]. The peak at 1157cm-^1^ is allocated to the ester bond (C–O–C) stretching vibration [12]. The absorbance peaks at 3024 cm^−1^ can be assigned to O–H stretch in carboxylic acid. The broad absorbance band at 3229 cm^−1^ can be attributed to the formation of hydrogen bonds among carboxyl groups.

Figure 2b displays the XRD models of the prepared P(St-BA-AA) collodal microspheres. The obvious diffraction peak at the position 2θ corresponding to 20° implies that the as-synthesized P(St-BA-AA) microspheres are amorphous in characteristics [20, 29].

Figure 2c displays the TGA result that was generated on the as-synthesized P(St-BA-AA) samples at a heat rate of 10 °C/min. The spectra unveiled no loss in weight from 0 °C to 224 °C for both terpolymer microspheres. Notwithstanding, there was a small reduction in weight (16.74%) as the temperature surpassed 224 °C. This could be ascribed to the depletion of water and carbon (IV) oxide from the ester and acrylate portion of the terpolymer samples. As the heating temperature rose past 383 °C, the rate of combustion of the synthesized terpolymer samples further increased till the temperature attained 440 °C. This result, consequently, revealed that both terpolymer microspheres were completely burnt at a heating temperature of 440 °C.

Figure 2d shows the glass transition temperature (Tg) of the as-synthesized P(St-BA-AA) samples. The result placed the Tg of both terpolymer colloidal samples at about 110 °C. This is an indication of that the prepared terpolymer microspheres have good thermal stability as far as it is applied within the aforementioned transition temperature. The observed Tg is higher than the established Tg values for PS [32]. This difference may be due to the interactions taking place in the P(St-BA-AA) microspheres, between the acrylate/acrylic molecules and polystyrene chains. The broad peak region of the spectra suggests that the melting point of the microspheres occurs over a temperature range of 186–218 °C. This may be due to the amorphous nature of the P(St-BA-AA) samples.

**Figure 3:**
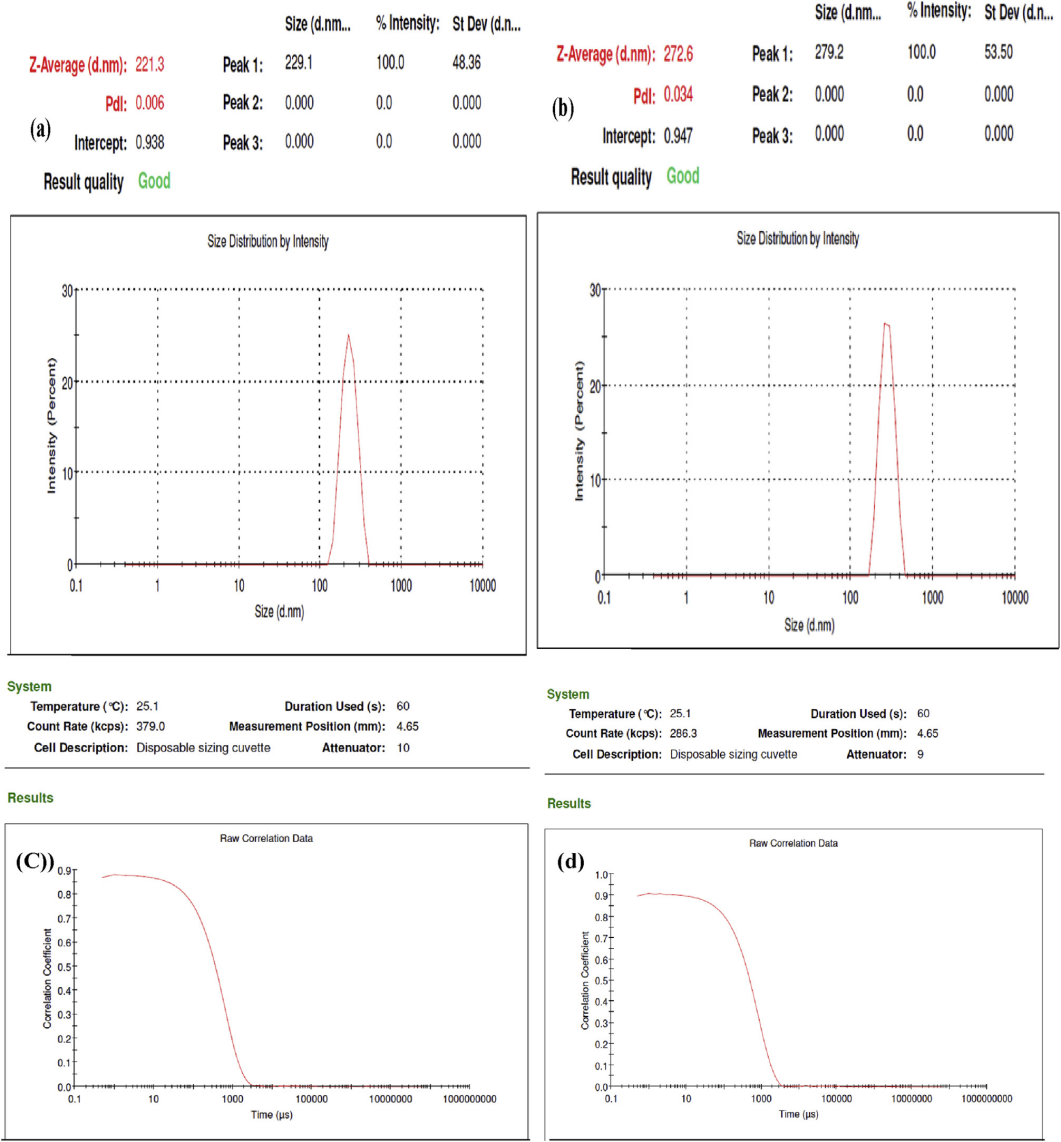
(a & b**)** Size distribution report by intensity and (c & d) correlogram of P(St-BA-AA)_1_ and P(St-BA-AA_)2_ under different initiator concentrations.

The particle diameters of the P(St-BA-AA) colloidal samples was regulated by varying the initiator concentration while leaving other reaction parameters fixed. Figure 3 shows the size distribution report by intensity and correlogram of the synthesized P(St-BA-AA)_1_ and P(St-BA-AA_)2_ microspheres under different initiator concentrations. Table 1 shows the consequence of increasing the initiator concentration from 0.36 mmol to 0.44 mmol on the average particle diameter of the fabricated terpolymer microspheres.

**Table 1:** Particle size regulation of P(St-BA-AA) microspheres.

	P(St-BA-AA)_1_	P(St-BA-AA)_2_
Average particle diameter (nm)	272.6	221.3
Polydispersity index (PDI)	0.034	0.006
Zeta potential (mV)	-35.60	-36.8
Initiator conc.(mmole)	0.36mmol	0.44mmol

The average particle diameter of the P(St-BA-AA)_1_ and P(St-BA-AA_)2_ microspheres was observed to be 272.6 nm and 221.3 nm respectively (Table 1). The correlogram result shows that the analysis is of good quality (Figure 3(c & d)). The result revealed a reduction in the average particle diameter from 272.6 to 221.3nm as the initiator concentration increased. This result is consistent with reports by previous studies [14, 29, 33, 34]. The increased polymerization rate brought about by the increase in initiator concentration may have led to a shorter nucleation period which resulted in a decrease in the particle size of the terpolymer microspheres. The observed dispersity index (PDI) of 0.034 and 0.006 is indicative of a narrow size range distribution for the synthesized P(St-BA-AA)_1_ and P(St-BA-AA)_2_ colloidal samples [35].

Zeta potentials of -35.6 mV and -36.8 mV observed in Table 1 and Figure 3 shows good stable colloidal dispersion of both synthesized terpolymer microspheres [30, 35].

### 3.3 Photonic crystals

**Figure 4:**
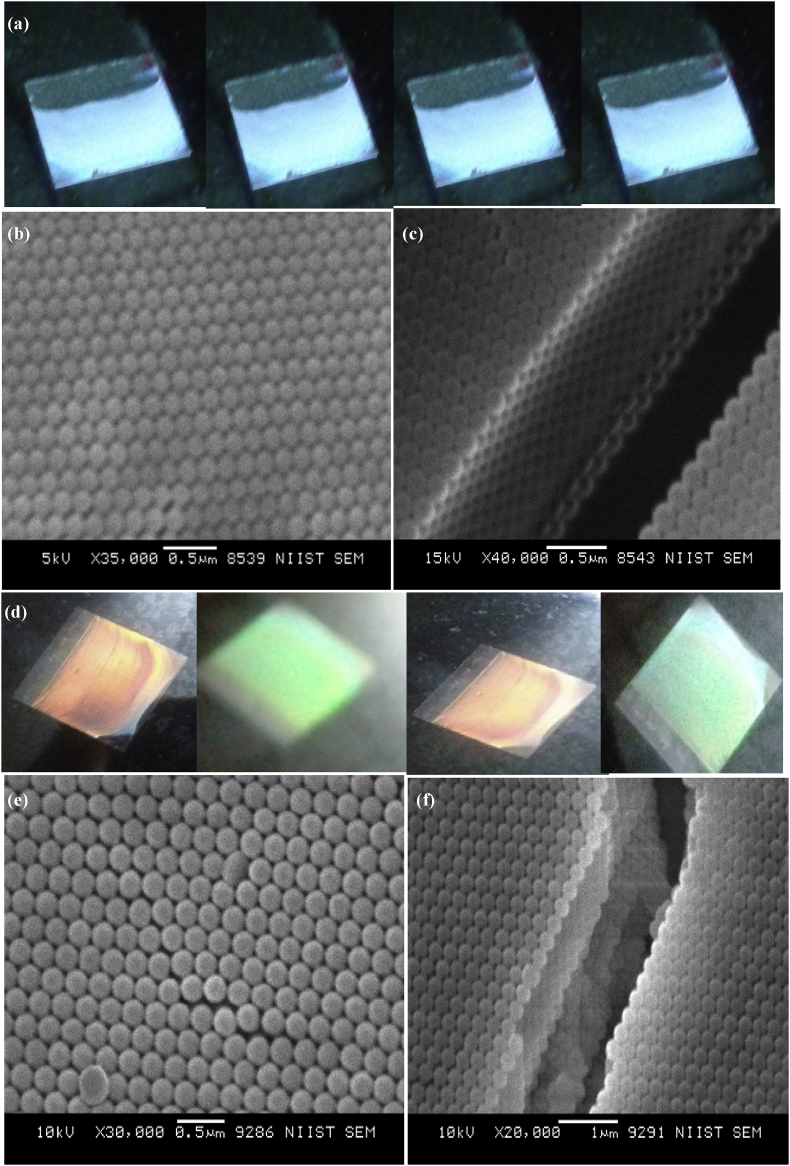
Coloured and SEM images of (a, b & c) P(St-BA-AA)1 and (d, e & f) P(St-BA-AA)2 photonic crystals (PCs).

Coloured photonic crystals (PCs) were fabricated via evaporative-assisted self-assembly process from the prepared P(St-BA-AA)_1_ and P(St-BA-AA)_2_ microspheres apiece. The generated photonic crystals from P(St-BA-AA)_1_ microspheres displayed a brilliant blue monochromatic colour (Figure 4a) while the colour obtained from P(St-BA-AA)_2_ photonic crystals changed from red to green with viewing angle (Figure 4d). The colours were attributed to the scattering and diffraction of light that occurs as a result of the combined effects of the good level of dispersity, intrinsic small sizes and the well-ordered arrangements of particles of both terpolymer samples [36].

Apart from the periodic arrangement of the as-synthesized P(St-BA-AA) colloidal particles, the size distributions of the colloidal particles can also influence the intensity of the colours that can be generated from a colloidal crystals. From our previous studies on colloidal crystals [12, 29], it has been established that a more uniform distribution of particle sizes always produces colour with higher intensity. The contrasts in colour between both synthesized P(St-BA-AA) colloidal crystals cannot be seen easily when inspected with the naked eyes.

Interestingly, when the photonic crystals were viewed through a scanning electron microscope, a three dimensional close-packed hexagonal periodic structures with multi-layer like arrangement of spherical shaped particles were observed (Figure 4(b, c, e & f)). It has been established that the closed packed crystal arrays shown by these structures are thermodynamically favoured by their low minimum Gibbs free energy [37, 38]. Crack defects between the domains of the photonic crystals films were observed (Figure 4(c & f)). This may be associated with liquid evaporation during the growing process of the crystals.

**Figure 5:**
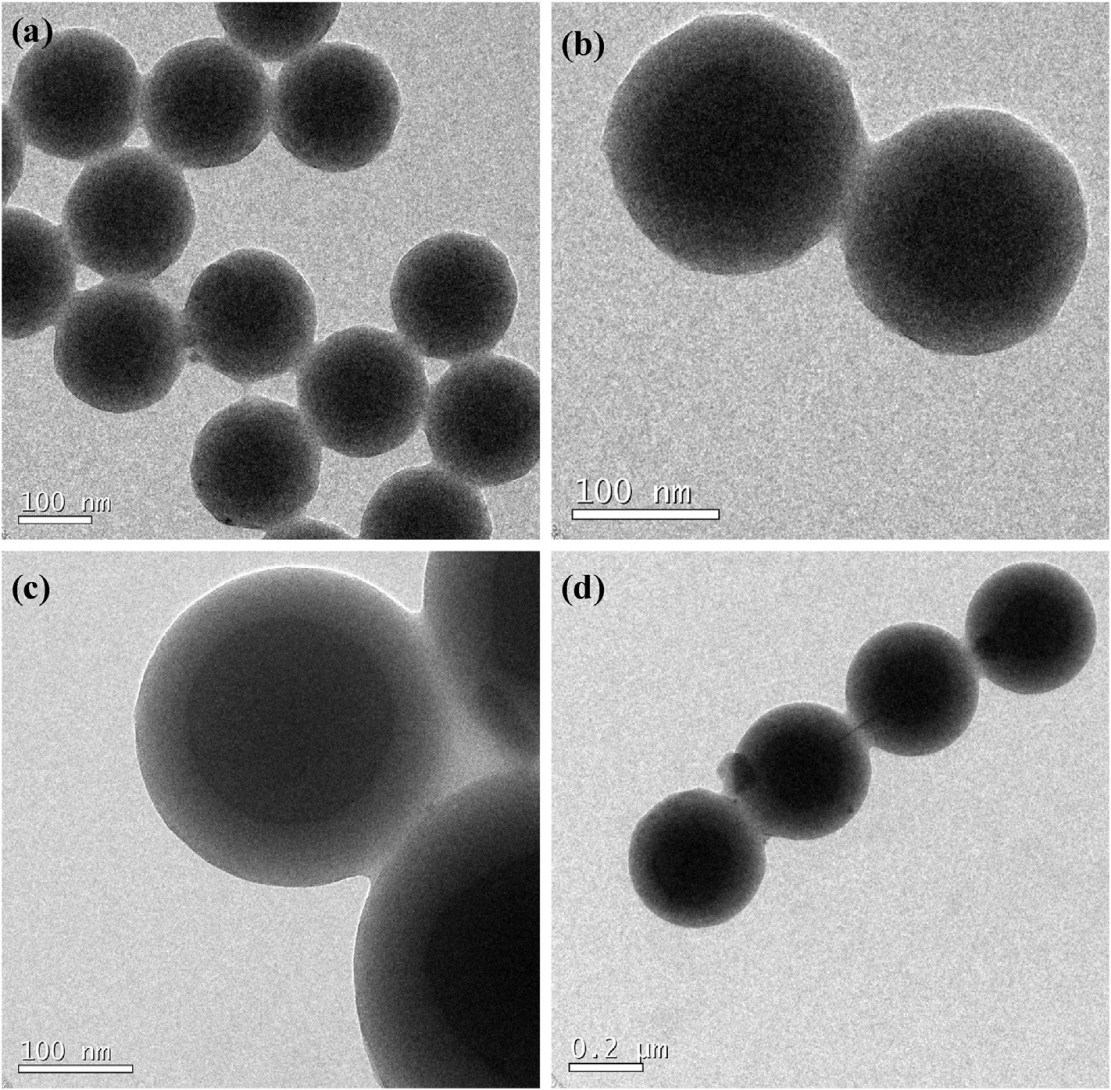
TEM images of core-shell (a & b) P(St-BA-AA)_1_ and (c & d) P(St-BA-AA)_2_ microspheres.

The Transmission electron micrographs of the prepared colloidal particles showed a core-shell structure (Figure 5(a, b, c & d)). This kind of structure has been established by previous studies [24, 30]. Core and shell sizes of 198/50nm and 176nm/30nm were respectively obtained for P(St-BA-AA)_1_ and P(St-BA-AA)_2_ samples. The TEM analysis placed the average particle diameter of the respective terpolymer microspheres at about 248nm and 196 nm while SEM analysis gave average particle diameters of 250 nm and 199 nm respectively.

The obtained sizes were also in agreements with the results obtained by dynamic light scattering (DSC), scanning electron microscope (SEM) and atomic force microscope (AFM) analysis.

**Figure 6:**
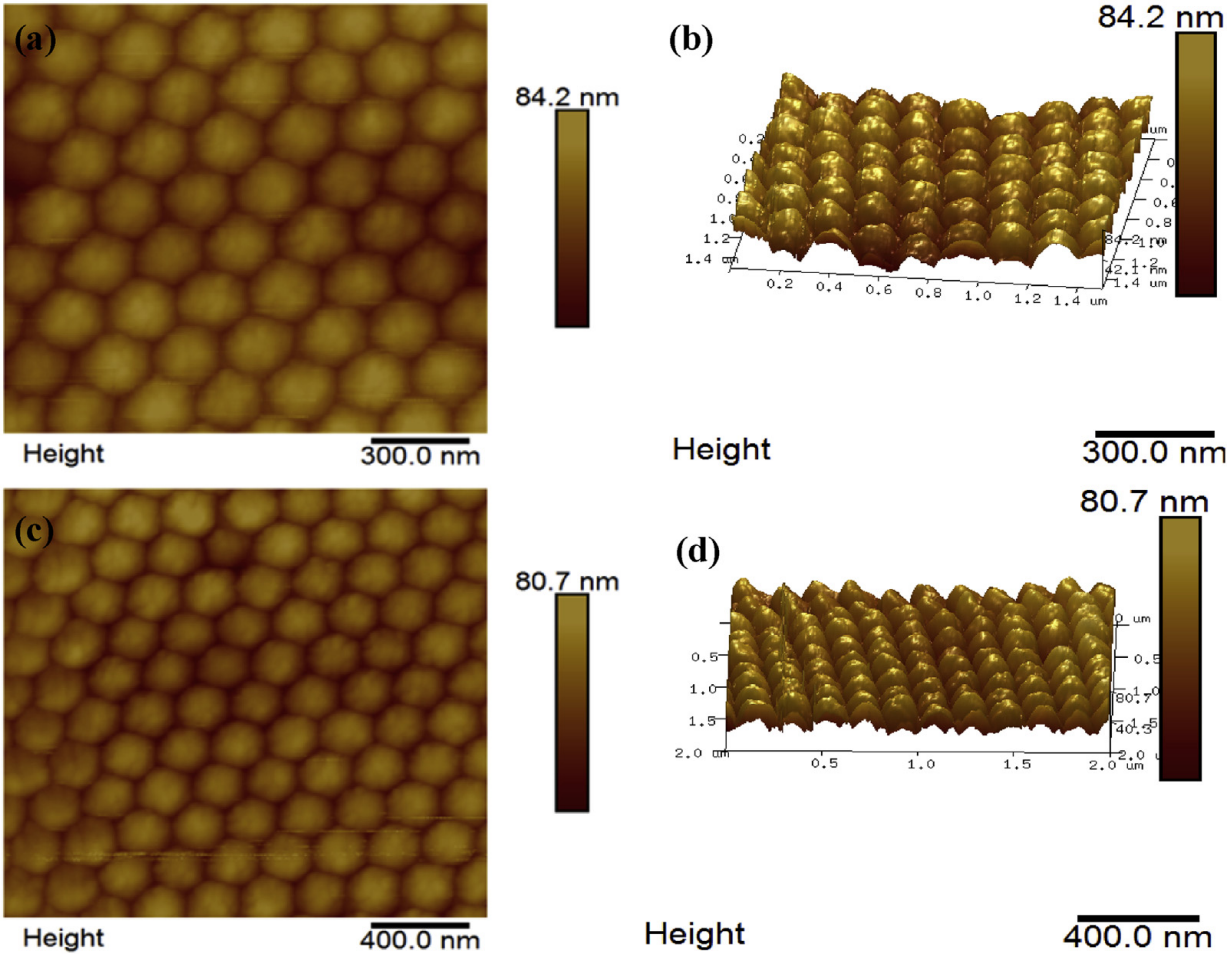
2D and 3D AFM images of core-shell (a & b) P(St-BA-AA)_1_ and (c & d) P(St-BA-AA)_2_ photonic crystals.

Figure 6 shows two and three-dimensional (2D & 3D) atomic force microscopic images (AFM) of the fabricated colloidal crystals. The AFM micrographs show an ordered arrangement of spherical shaped monodisperse particles. The obtained morphology conforms to the hexagonal compact morphology obtained by the scanning electron micrographs shown in Figure 4(b, c, e & f). The average particle diameter, particle height and surface roughness were placed at about 198nm, 26nm, 9.31nm for the P(St-BA-AA)_1_ microspheres (Figure 6(a & b)) and 252nm, 30nm, 10.3nm for the P(St-BA-AA)_2_ microspheres (Figure 6(c & d)) respectively.

### 3.4 Printing of P(St-BA-AA) colloidal ink on ink-jet paper surface

P(St-BA-AA)_1_ and P(St-BA-AA)_2_ microspheres were utilized in the formulation of UV curable P(St-BA-AA))_1_ and P(St-BA-AA))_2_ colloidal inks respectively. Thereafter, both prepared colloidal inks were transferred onto the surface of untreated inkjet-paper substrates with the aid of a pipette at room temperature. The colloidal mixture was maintained at the deposition points, where they were allowed to dry under UV irradiation. The photo-polymerization of Acrylic Amide (AAm) into the interstices of the colloidal particles took place after two minutes (Figure 4(a & d)). This fast-drying rate could be ascribed to the combined effects of Acrylic amide (AAm) and the photoinitiator added during the ink formulation.

**Figure 7:**
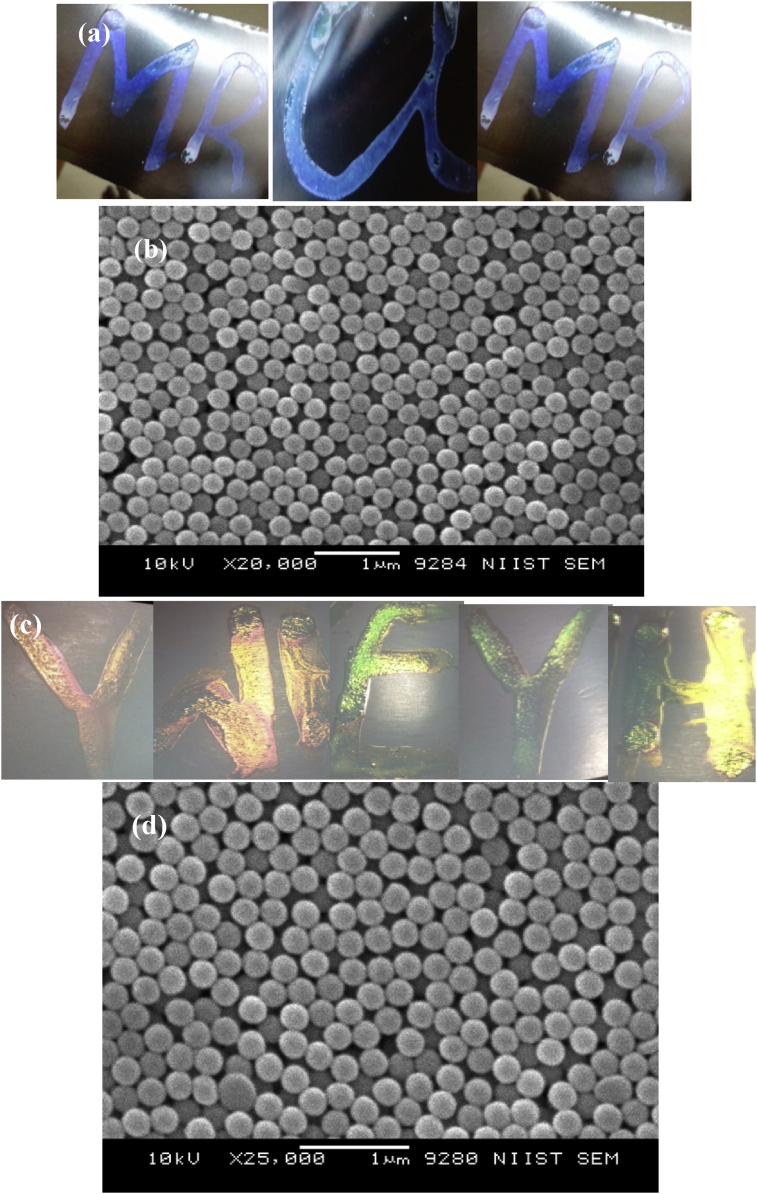
Coloured and SEM images of (a & b) P(St-BA-AA)_1_ and (c & d) P(St-BA-AA)_2_ photonic crystals ink microdots generated on inkjet paper substrate.

Figure 7 shows (a & c) coloured photonic crystal microdots that were obtained by manually printing P(St-BA-AA) colloidal inks on inkjet-paper substrates. The printing was carried out without any specialized tools or substrate preparation. A blue colour which did not change with variations in observation angle (monochromatic) was observed for the photonic crystals generated by the prepared P(St-BA-AA)_1_ colloidal ink. This colour observation was quite different from the P(St-BA-AA)_2_ colloidal ink photonic crystals films which displayed a red colour which varied to green with observation angle. The colour of the formulated P(St-BA-AA)_1,2_ colloidal inks (Figure 7(a & c)) and the P(St-BA-AA)_1,2_ photonic crystals (Figure 4(a & d)) were observed to behave similarly.

Figure 7(b & d) shows the scanning electron micrographs (SEM) generated from the colloidal crystal microdots by using P(St-BA-AA) colloidal printing inks on inkjet-paper substrates. The P(St-BA-AA) microspheres formed spherical shaped particles with non-compact periodic structures, leading to highly ordered, crystalline colloidal crystals. However, the assembly morphologies obtained from both P(St-BA-AA) colloidal spheres (Figure 4(b, c, e & f)) were observed to be more compact when compared to the P(St-BA-AA) colloidal printing ink (Figure 7(b & d)). A previous study has shown that non-compact colloidal crystal arrays can be applied in photonic materials for some specific applications because they exhibit a wider range of band-gap compared to close-packed arrays [39]. The average particle diameters of the P(St-BA-AA) colloidal particles were observed to be different from the P(St-BA-AA) colloidal ink particles after photo-polymerization. The particle size increased from 250nm to 276nm and 197nm–233nm for P(St-BA-AA)_1_/P(St-BA-AA)_2_ microspheres and their respective ink formulations. This is an indication that the Acrylic Amide (AAm) added during the ink formulation, has been completely incorporated into the P(St-BA-AA) colloidal particles.

**Figure 8:**
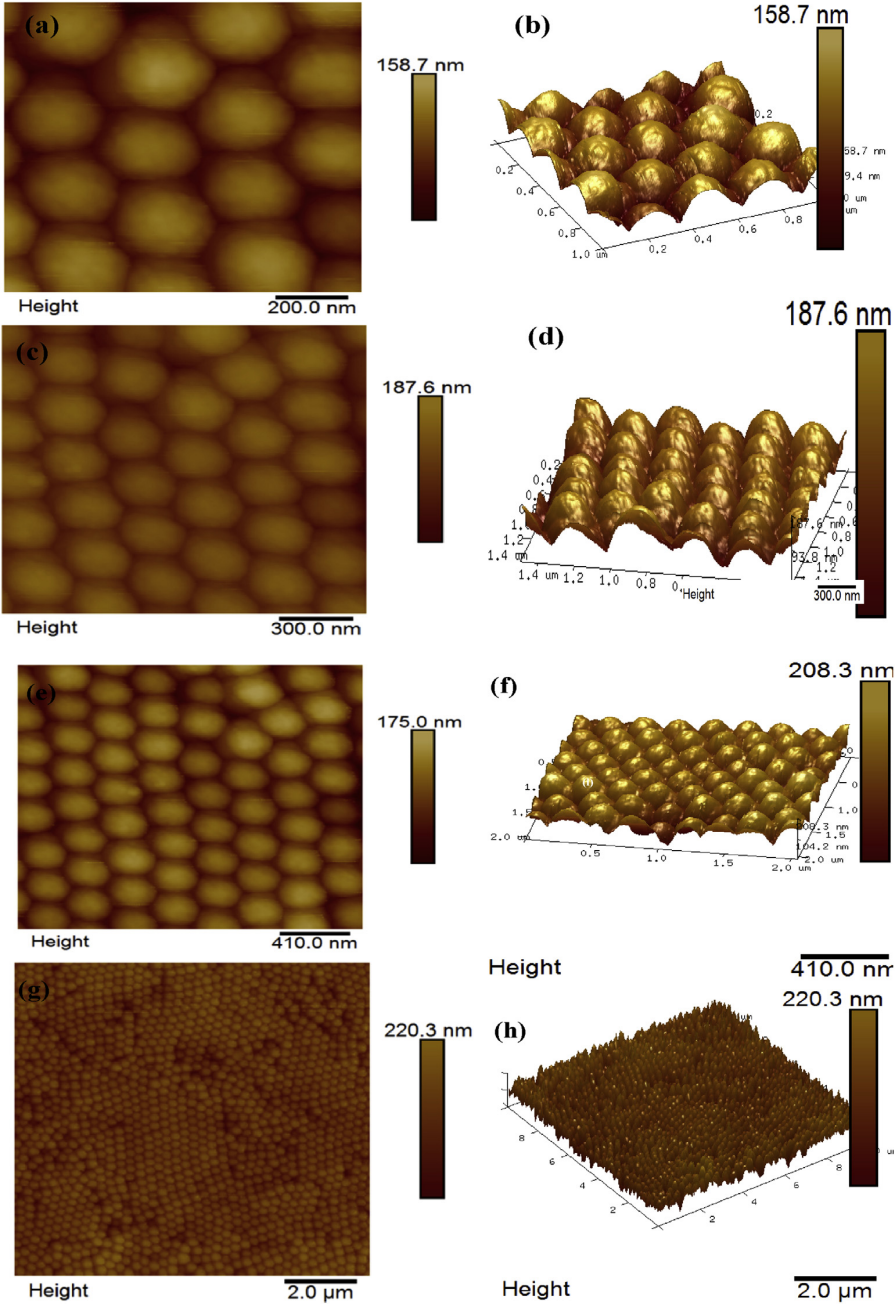
Atomic force microscopic images of (a, b, c & d) P(St-BA-AA)_1_ and (e, f, g & h) P(St-BA-AA)_2_ colloidal ink crystals.

Figure 8 shows two and three dimensional atomic force microscopic (AFM) images of the P(St-BA-AA) photonic crystals ink microdots fabricated on inkjet-paper substrates. An ordered monodispersed spherical shaped particles similar to the morphology revealed by the scanning electron micrographs shown in Figure 4(b c, e & f) was observed. The average particle diameter, peak height and surface roughness of the P(St-BA-AA)_1_ microspheres are 253nm, 23.3nm and 52.5nm respectively.

## 4 Conclusion

In this article, we have generated photonic crystal microdots from monodispersed core-shell P(St-BA-AA) microspheres and their colloidal ink formulation on glass slide and ink-jet paper respectively. They were both assembled into a well ordered arrangement of core-shell spherical shaped colloidal crystal microdots. However, the terpolymer microspheres spheres retained a three dimensional close-packed hexagonal periodic assembly with multifaceted layers. This effortless technique of fabricating colloidal crystal arrays with eccentric colour properties on ink-jet paper may find practical applications in security, optical devices, environmentally friendly color coatings, and decorative materials.

## Declarations

### Author contribution statement

Ikhazuagbe Hilary Ifijen: Conceived and designed the experiments; Performed the experiments; Analyzed and interpreted the data; Wrote the paper.

Esther Uwidia Ikhuoria: Conceived and designed the experiments; Analyzed and interpreted the data.

### Funding statement

This work was supported by TWAS.

### Competing interest statement

The authors declare no conflict of interest.

### Additional information

No additional information is available for this paper.
